# The Relations Between Sensory Modulation, Hyper Arousability and Psychopathology in Adolescents with Anxiety Disorders

**DOI:** 10.3390/children12020187

**Published:** 2025-02-05

**Authors:** Ginan Hammud, Ayelet Avital-Magen, Hiba Jabareen, Reut Adler-Tsafir, Batya Engel-Yeger

**Affiliations:** 1 Department of Occupational Therapy, Faculty of Social Welfare and Health Sciences, University of Haifa, Haifa 3498838, Israel; ghammud@campus.haifa.ac.il (G.H.); reutadler@gmail.com (R.A.-T.); 2 Child and Adolescent Mental Health Clinic, Haemeq Medical Center, Afula 1834111, Israel; ayelet_av@clalit.org.il (A.A.-M.); hibajaba@clalit.org.il (H.J.)

**Keywords:** anxiety disorders, sensory modulation, hyper-arousability, daily life

## Abstract

Background: Sensory modulation may play a significant role in psychiatric conditions, including anxiety, and explain arousability levels, behavioral disorders, and functional deficits. Yet, studies about sensory modulation in adolescents with anxiety disorders are scarce. Purpose: To profile the prevalence of sensory modulation difficulties (SMDs) in adolescents with anxiety and examine their relations to arousability and psychopathology. The study compared adolescents with anxiety disorders to healthy controls using physiological measures and self-reports that reflect daily life scenarios. Then, the study examined the relationship between SMDs, arousability, and psychopathological severity in the study group. Method: Participants were 106 adolescents, aged 10.5–18 years and their parents. The study group included 44 participants diagnosed with anxiety disorder by psychiatrists. The control group included 62 healthy participants matched by age and gender to the study group. Parents completed the demographic questionnaire and the Child Behavior Checklist (CBCL). The adolescents completed The Revised Children’s Manifest Anxiety Scale (RCMAS) and the Adolescent/Adult Sensory Profile (AASP) and underwent the electrodermal activity (EDA) and pulse rate tests while listening to extreme sensory stimuli of auditory startles. Results: Based on AASP, the study group had a higher prevalence of SMDs expressed in lower sensory seeking, difficulties in registering sensory stimuli, and higher sensory sensitivity and avoidance. The study group presented higher arousability while listening to the startles as manifested in higher heart rate and EDA responses. The physiological results correlated with SMD levels measured by the AASP self-reports. SMDs correlated with psychopathological severity. Conclusions: SMDs may characterize adolescents with anxiety disorders and impact their arousability, symptoms severity, and daily functioning. Therefore, sensory modulation should be evaluated using both self-reports (to reflect implications in real life from patients’ own voices) along with objective measures to explain daily behaviors by underlying physiological mechanisms. This may focus intervention towards better health, function, and development.

## 1. Introduction

Anxiety disorders are prevalent among 20.5% of children and adolescents around the world [1]. As outlined in The Diagnostic and Statistical Manual of Mental Disorders (DSM-V), anxiety disorders include social anxiety disorder (SAD), specific phobias, generalized anxiety disorder (GAD), panic disorder (PD), separation anxiety, selective mutism, and agoraphobia. Among children and adolescents under the age of 18, the definition of anxiety disorder is relevant if symptoms last for at least six months [2]. Anxiety disorders often coexist with other psychiatric conditions, like attention deficit hyperactivity disorder (ADHD), major depression, somatic symptom disorders, personality disorders, and substance abuse disorders [3,4]. The symptoms of anxiety disorders include worry about the future, obsessive and repetitive thoughts, and excessive fear [2,5] that physiologically are manifested in hyper-arousability and elevated sympathetic activity (increased heart rate, shortness of breath, sweating, tremor, chest pain, nausea, etc.). Hyper-arousability may be triggered by a disproportionate response to stimuli from the environment that are perceived as threatening [6]. This may explain the high prevalence of sensory modulation difficulties (SMDs), mainly sensory hypersensitivity in patients with anxiety disorders [7,8,9,10].

*Sensory modulation* refers to the ability of the central nervous system to modulate and organize sensory input in a graded manner according to environmental demands [11]. Difficulties in sensory modulation result in hypersensitivity or hyposensitivity and lead to unmodulated behavior and malfunction. One main model that refers to the implications of sensory modulation on behavior is Dunn’s model. According to this model, the interaction between the neural threshold for sensory input and the strategies used for coping with the neural threshold (active/passive) defines behavioral and functional outcomes [11]. This interaction results in four profiles: sensory sensitivity (low neural threshold and passive strategy)—people who do not actively eliminate unpleasant stimuli; sensation avoidance (low neural threshold and an active strategy)—people who actively avoid unpleasant stimuli, such as avoiding walking on sand; low registration (high neural threshold and passive strategy)—people who need extensive intensity of sensory stimuli to recognize it, but do not actively seek rich sensory input (as sports activity), therefore, may miss information, and be less attentive; sensory seeking (high neural threshold and an active strategy)—people who actively seek for intense stimuli in their environment, as going to parties; coloring their rooms; do extreme sport [12]. One of the gold standard measures for sensory modulation is the Sensory Profile [13]. Based on the sensory profile, SMDs are prevalent among 57% of children with psychiatric disorders [14]. The significant benefit of the sensory profile is that it reflects SMDs as expressed in behavioral responses to sensory input in daily life scenarios.

Indeed, when the ability to modulate sensory information is impaired, it affects the individual’s ability to properly perceive the environment and function in it. Therefore, anxiety and hyper-arousability may be elevated, as manifested in objective physiological measures such as electrodermal activity (EDA) [15], higher cortisol levels [16], and elevated heart rate [17,18,19,20]. In children, anxiety symptoms were manifested in physiological measures such as over-reactive skin conductance (EDA) [21]. Hence, the objective and subjective measures may explain unmodulated behaviors such as “fight or flight,” impulsivity, distraction, nonadaptive behavior and daily dysfunction [22].

Hence, the complexity of the symptoms of anxiety disorders, the prevalent comorbidities of SMDs as well as their severe consequences on a child’s/adolescent’s daily life, serve the rationale for studying the interrelation between these factors in depth in order to improve intervention. This knowledge is especially critical in adolescence where development is essential for determining later human life and wellbeing. Therefore, this study aimed to (1) profile sensory modulation of adolescents with anxiety disorders, as compared to healthy controls, using objective and subjective measures and (2) among the study group, examine the relationship between sensory modulation and anxiety psychopathology.

Study hypotheses: (1) adolescents with anxiety disorders would have a significantly higher prevalence of SMDs as expressed in self-reports and in objective measures of sympathetic hyper-arousability than healthy controls; (2) among the study group—(a) SMDs according to self-reports would correlate with the objective manifestations and (b) SMDs would significantly correlate with severe psychopathology.

## 2. Materials and Methods

### 2.1. Participants

This study is a cross-sectional and correlative study. Based on the power calculation of MANOVA global effects, with f2 = 0.07 at a significance level of *p* = 0.05 and power = 0.80, this study was supposed to include 142 adolescents. However, due to various constraints, this study included 106 adolescents. The participants’ age range was 10.5–18 years. The study group included 44 adolescents, 21 boys and 23 girls, with a mean age of 13.69 (SD = 1.99), diagnosed with anxiety disorder by psychiatrists, according to the DSM-5 and a clinical interview. All participants of the study group were patients of the Child and Adolescent Mental Health Clinic (blinded). The control group included 62 healthy adolescents, 33 boys and 29 girls, mean age = 13.39 (SD = 1.99), with normal development and emotional health, based on their parents’ report and their Child Behavior Checklist (CBCL) normal scores (Achenbach and Rescorla, 2001) [23]. The study groups were matched by age (t = −0.76, *p* = 0.80) and gender (χ^2^ (1) = 0.31, *p* = 0.57) but significantly differed in mother’s education (χ^2^ (1) = 15.66, *p* < 0.001) and in socioeconomic status (χ^2^ (2) = 21.14, *p* < 0.001) (see Table 1 and Table 2). Mothers’ education and socioeconomic status parameters were significantly positively correlated. Therefore, statistical adjustments were performed and the mother’s education was held as a covariant. Exclusion criteria: adolescents with unstable severe health/chronic illnesses who are taking pharmacological treatment that has a detrimental effect on the nervous system or addictive substances (beyond the psychiatric medications in the study group) and those with uncorrected sensory impairment (e.g., by glasses).

### 2.2. Measures

#### 2.2.1. Subjective Measures—Self-Reports

##### Completed by Parents

A sociodemographic questionnaire.

The Child Behavior Checklist (CBCL) [23] was used to profile psychopathological symptoms. This standard measure screens behavioral and emotional problems in children and adolescents ages 4–18. This measure includes two sections: 20 items that measure social functioning and 113 items that measure emotional–behavioral problems (behavioral profile). For this study, the behavioral part was used. Answers are marked on a 0–2 scale (0 = not true for my child, 1 = somewhat/sometimes true, and 2 = very true or often true). Parents rate the items of problems based on the past six months. Raw scores are converted into T scores that indicate whether the participant exhibits abnormal behaviors with respect to norms by age and gender. A clinical cross-sectional score above 63, based on the total T-score, indicates clinical functioning, while scores between 60–63 suggest borderline functioning and scores below 60 are considered within the normal range. This measure demonstrates high test-retest reliability of 0.9, strong internal consistency of 0.72–0.97, and good criterion validity [23].

##### Completed by the Adolescents

The Revised Children’s Manifest Anxiety Scale (RCMAS) [24] aims to profile anxiety levels in children and adolescents aged 6–19 years. The RCMAS includes 37 items, divided into three scales: The Physiological Scale consists of 10 items related to physical symptoms of anxiety, such as sleep problems, stomach issues and breathing difficulties. The Worry and Oversensitivity Scale consists of 11 items related to excessive, unfocused worrying and concern about external stressors. The Social Concern and Concentration Scale includes 7 items related to fears of social isolation and attention difficulties. The remaining 9 items comprise The Lie Scale, which measures social desirability and “fake good”. Each item describes an experience, respondents note “Yes” or “No”. If the response is “Yes” it indicates the presence of an anxiety symptom and it receives a score of 1. If the response is “No” it indicates the absence of an anxiety symptom and it receives a score of 0. The total score is calculated based on the three scales of the RCMAS which consist of 28 of the 37 items (the 9 items of the lie scale are excluded). Higher scores indicate higher levels of anxiety.

The RCMAS has good psychometric properties [24,25]. According to the standardization sample data [24], a T-score of 60 is equivalent to the cut-off score of 19 (M = 11.70, SD = 6.21).

Adolescent/Adult Sensory Profile (AASP) Questionnaire [26]: This 60-item self-report measures sensory modulation among individuals aged 11 and above. Respondents rate the frequency of their behavioral reactions to sensory experiences in everyday life (for example: “I’m uncomfortable wearing certain fabrics”; “I avoid elevators and/or escalators because I dislike the movement”). Answers are scored on a 1–5 Likert scale (1 = almost never, 5 = almost always). The higher the score, the more extreme the sensory modulation pattern. The AASP has four outcome measures in accordance with the four quadrants of Dunn’s model (see above): low registration, sensory seeking, sensory sensitivity, and sensation avoidance (15 items in each category). Five categories are identified in each sensory modulation pattern: “much less than most people,” “less than most people,” “similar to most people,” “more than most people,” and “much more than most people” [26]. In the present study, the five categories were combined into three: (1) “less than most people”—that is, one to two standard deviations below the norm, (2) “similar to most people”—within the norm, and (3) “more than most people”—one to two standard deviations above the norm. Sensory modulation norms exist for different age groups (11–17, 18–64, 65 and over). SMDs are defined for participants found in the “less” or “more than most people” ranges. Internal reliability was found to range from 0.64–0.78 [26]. Discriminant validity was found between groups in the psychiatric population [27].

#### 2.2.2. Objective Measures

In order to reflect the physiological manifestations of SMDs, and based on the literature on SMDs, the following measures examine arousability/sympathetic reactivity to sensory stimuli of auditory startles:

An electrodermal activity test (EDA) [28] measures the skin conductivity (in units of micro-siemens) due to the activity of the sweat glands in the skin in response to a stressogenic stimulus. Two 5 mm electrodes (Mindlife, Jerusalem, Israel) are connected to the second and fourth fingertips of the non-dominant hand using a Velcro strap. The electrodes are connected to a sensor, receiver, and amplifier, providing 10 samples per second [28]. The experiment is divided into three phases, while the subject is sitting in front of a computer screen watching kaleidoscope shapes in order to neutralize stimuli distractions from the environment and to increase compliance which is often used in psychophysiology research [29]. The first phase lasts a minute (for acclimatization). In this phase, the subjects hear white noise through the headphones. The second phase—the startles session—includes the auditory startles which appear randomly, 40 times, for 3 min, at a volume of 90 dB with each stimulus lasting 30 ms. In the third and final phase, the relaxation session, white noise is heard again for one minute (allowing adjustment). The experiment lasts 5 min in total.

The outcome measures of the EDA test are (1) reaction time (in seconds) and amplitude height in response to the first startle; (2) time (in seconds) and height of the maximum amplitude during the 3 min of auditory startles—to measure maximal arousability; (3) the following measures reflected adaptation: time and height of minimal amplitude during the 3 min of auditory startles session; and (4) time and height of maximum and minimum amplitude at the time of relaxation session (third session).

A pulse oximeter (Contec Systems CMS50N; Qinhuangdao, China) is an additional measure of sympathetic activity reflected in heart rate in response to auditory startles. The pulse oximeter was attached to the other finger of the hand that is not connected to the EDA test during all EDA sessions.

### 2.3. Procedure

After obtaining ethics approval from the [blinded] and from the Helsinki Committee [blinded], adolescents and their parents from the study group were recruited via advertisements published at the Child and Adolescent Mental Health Clinic [blinded]. Participants in the control group were recruited through similar advertisements published in neighborhoods in the same geographic area.

**Figure 1:**
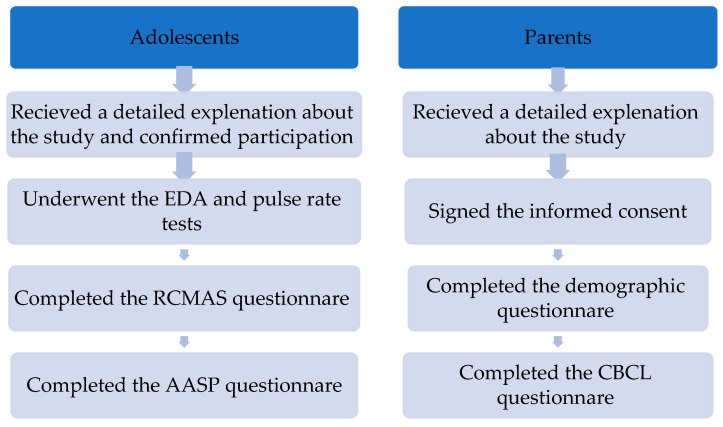
Experimental schedule completed by the participants and their parents.

Parents interested in participating with their children in this study were contacted by the study conductor and a meeting was scheduled with them at the clinic (study group) or at their homes (control group). In the meeting, the participants and their parents received a detailed explanation about the study and observing ethical rules declaration. The parents signed the informed consent form and completed the demographic questionnaire and the CBCL. Adolescents underwent the EDA test and the heart rate measurement. Then, they completed the RCMAS and the AASP with the study conductor (See Figure 1).

All questionnaires were anonymous. Each questionnaire received a serial number without identifying details of the participants and the data was stored in a protected folder on the researcher’s computer and all paper data was kept in a locked closet.

### 2.4. Statistical Analysis

Data was analyzed using the Statistical Package for the Social Sciences (SPSS)-28. After analyzing descriptive statistics and testing normality, the data distribution was found to be normal in all study measures, except for the CBCL scores in the control group. Therefore, chi-square analysis examined the prevalence of SMDs according to the AASP norms, clinical emotional behavior problems according to the CBCL and anxiety problems according to the RCMAS cutoff scores. T-tests examined the differences between the groups in socio-demographic parameters. For examining the first hypothesis, a univariate analysis of covariance (ANCOVA) examined the differences between the study groups in total scores of the RCMAS and heart rate. A multivariate analysis of covariance (MANCOVA) examined the differences between groups in the subscales scores of the RCMAS, AASP, and EDA scores. Mother’s education was held as a covariate throughout the analyses. A Mann–Whitney test examined the differences between groups in CBCL scores. For examining the second hypothesis, the Pearson correlation test examined the correlations between the subjective (AASP) and objective (EDA and heartrate) measures and between sensory modulation and psychopathological symptoms severity according to the RCMAS and the CBCL. The significance level was *p* ≤ 0.05.

In our study, the control group is larger in terms of the number of participants than the study group due to difficulties in recruiting participants for the study group. Furthermore, some participants were missing part of the data (for example, one of the questionnaires), so the degrees of freedom varied accordingly. In the context of the EDA test, for example, one participant in the study group had no responses to the test, and six participants showed no response to the first stimulus.

## 3. Results

Before examining the study’s hypotheses, both groups were compared in their CBCL and RCMAS scores to verify inclusion criteria and provide the profile of each group.

### 3.1. The Differences in Emotional and Behavioral Modulation Psychopathology Between Both Groups According to the CBCL

**Figure 2:**
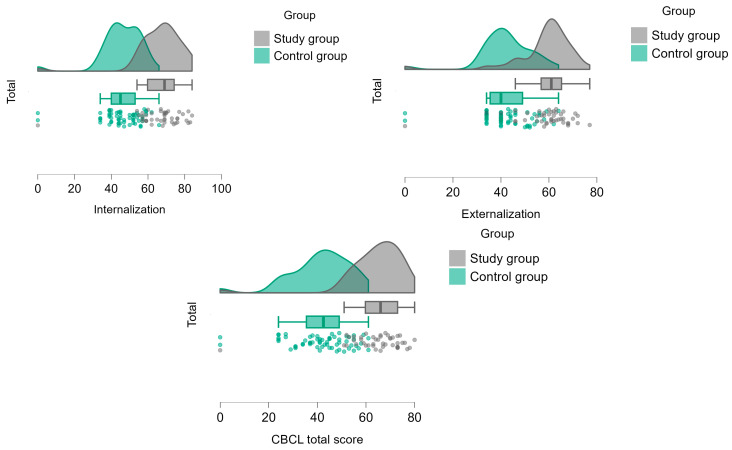
Differences between groups in CBCL scores.

According to the Mann–Whitney test, emotional and behavioral modulation problems were significantly more prevalent among the study group compared to the control group, as reflected in all CBCL scales and in the total score: the study group had greater difficulties in internalizing and externalizing symptoms. The study group had higher difficulties in anxiety symptoms, depression symptoms, and somatic complaints. Moreover, the study group showed significant behavioral problems such as social, thought and attention problems, rule-breaking, and aggressive behavior. (See Table 3, Figure 2)

According to chi-square analysis and the cutoff total CBCL score of 63, the study group had a significantly higher prevalence of clinical emotional and behavioral modulation problems (62.8%, CI95 48–76%) than the controls (0%, CI95 0–6%) (χ^2^ = 51.059, *p* < 0.001). The Fischer exact test indicated a significant difference between groups (*p* < 0.001).

### 3.2. The Differences in Anxiety Rates Between Both Groups According to the RCMAS

**Figure 3:**
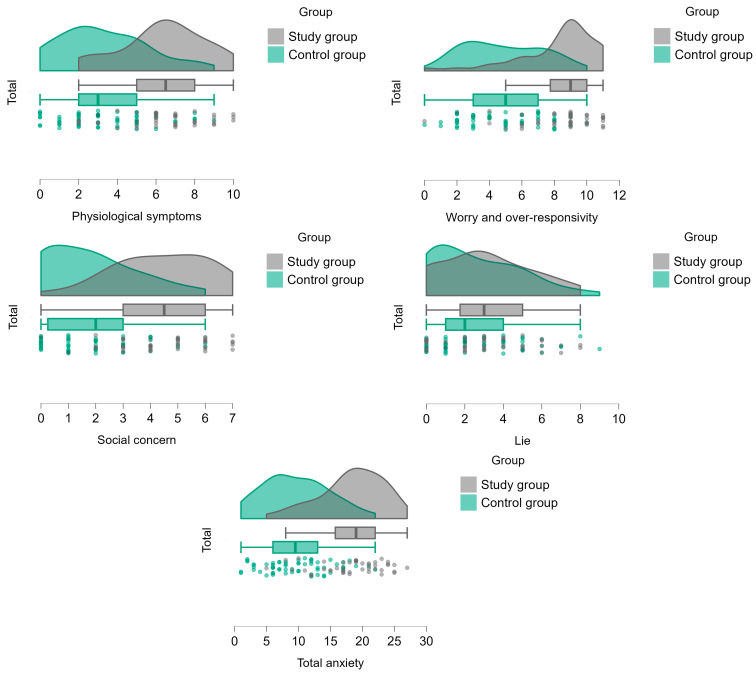
Differences between groups in RCMAS scores.

According to the MANCOVA and ANCOVA tests, anxiety symptoms were more prevalent among the study group, as reflected in all RCMAS scales: physiological, worry and over-responsivity, social concern, and concentration symptoms, except for the lie scale, and in total score, accordingly. The effect size for the observed anxiety symptoms variables was the partial eta square η_p_^2^ which ranged from 0.289 to 0.373, indicating large effect sizes (see Table 4, Figure 3).

According to chi-square analysis and the cutoff score of 19, the study group had a significantly higher prevalence of anxiety (54.5%, CI95 40.07–68.3%) than the controls (4.8%, CI95 1.66–13.29%) (χ^2^ = 33.496, *p* < 0.001). The Fischer exact test indicated a significant difference between groups (*p* < 0.001).

**Hypothesis** **1.** *Differences between the study groups in sensory modulation*.

### 3.3. Subjective Measure—The AASP Self-Report

**Figure 4:**
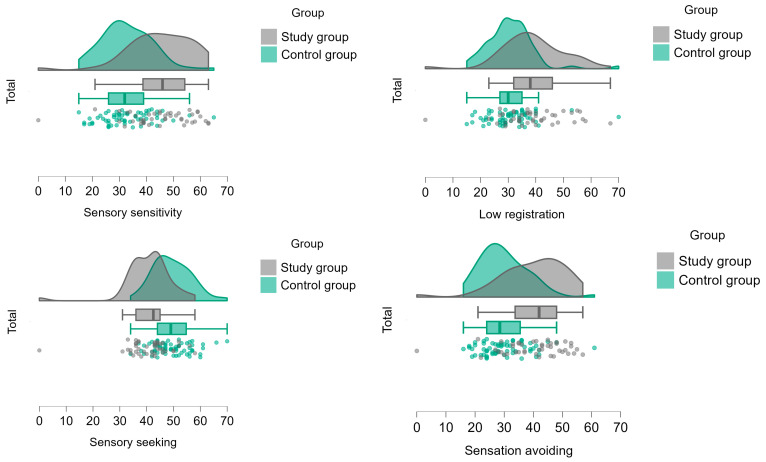
Differences between groups in AASP scores.

According to the MANCOVA test, a significantly higher prevalence of SMDs was found among the study group as manifested in the four AASP sensory profiles: adolescents with anxiety disorders had significantly greater sensory sensitivity and avoidance, lower ability to register sensory input, and lower tendency to seek sensory input. The effect size of the observed variables of SMDs was the partial eta square η_p_^2^ that ranged from 0.145 to 0.278, indicating large effect sizes (see Table 5, Figure 4). As presented in Table 6, according to chi-square analysis, this was also manifested in higher prevalence of abnormal SMDs (based on AASP normal/abnormal ranges) among the study group, which showed greater sensory sensitivity (χ^2^ = 26.025, *p* < 0.001), higher difficulty to register sensory input (χ^2^ = 21.328, *p* < 0.001), greater sensation avoidance (χ^2^ = 29.457, *p* < 0.001), and lower tendency to seek for sensory input (χ^2^ = 19.082, *p* < 0.001). The Fischer exact test indicated a significant difference between groups (*p* < 0.001).

### 3.4. Objective Measures–EDA and Heartrate

According to the MANCOVA test, greater reactivity to auditory startles was found in the EDA of the study group as compared to the controls. In response to the first stimulus, the study group had a significantly shorter reaction time and higher amplitude height. When referring to session 2 (the startles session) and 3 (the relaxation session), the study group had a significantly higher height of the maximum amplitude, reflecting higher arousability and difficulties in adapting and relaxing.

**Figure 5:**
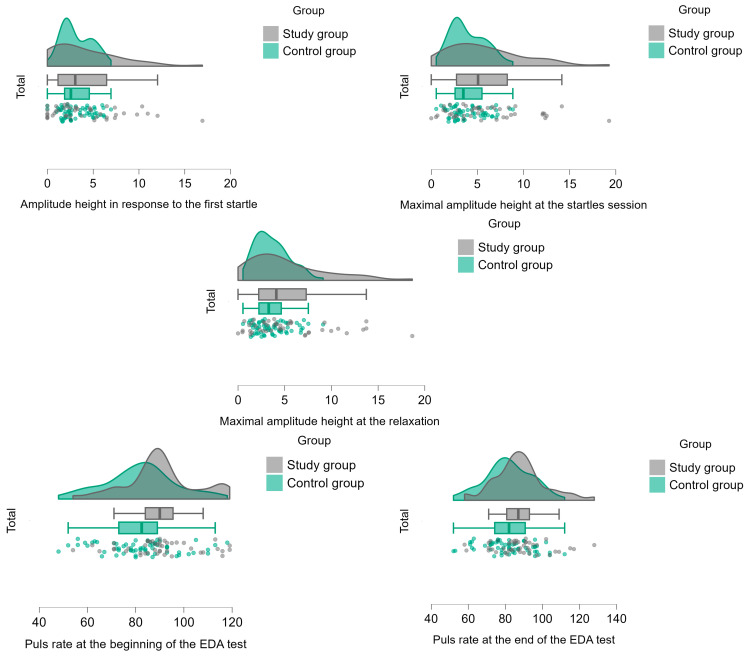
Differences between groups in EDA and pulse rate scores.

**Figure 6:**
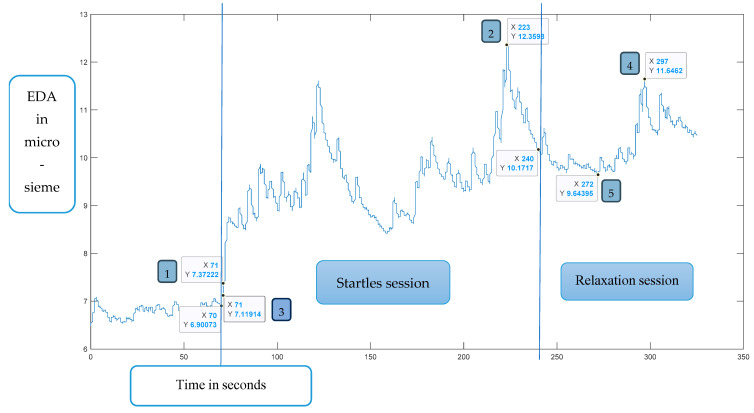
EDA recording of a study group participant. Note: 1 = reaction time and amplitude height in response to the first startle; 2 = time and height of the maximum amplitude during the 3 min of auditory startles; 3 = the time and height of minimal amplitude during the 3 min of auditory startles session; 4 = time and height of maximum amplitude at the time of relaxation session; 5 = time and height of minimum amplitude at the time of relaxation session.

According to the ANCOVA test, the study group also showed higher heart rate than the control group at the beginning and the end of the EDA test. The effect size of all the observed variables was the partial eta square η_p_^2^ that ranged from 0.048 to 0.386, indicating small to large effect sizes (see Table 7, Figure 5 and Figure 6). Nevertheless, no significant differences were found between the study groups in the heart rate delta (starting point and ending of the EDA procedure).

**Hypothesis** **2.** *Correlations between variables in the study group*.

### 3.5. The Correlations Between Subjective and Objective Measures

According to the Pearson correlation test, medium significant negative correlations were found between the AASP and the physiological manifestations—the EDA and heart rate. Lower sensory seeking correlated with higher amplitude height in response to the first startle (r = −0.327, *p* = 0.05), with the maximal (r = −0.415, *p* = 0.007) and the minimal amplitude (r = −0.458, *p* = 0.003) during the startles’ session as well as with the maximal (r = −0.332, *p* = 0.034) and the minimal amplitude (r = −0.355, *p* = 0.023) after the startles’ session—in the relaxation session. Lower sensory seeking also correlated with the time of the maximal amplitude (r = −0.340, *p* = 0.03) in relaxation and with elevated heart rate at the beginning of the EDA test (before starting the startles session) (r = −0.355, *p* = 0.034).

### 3.6. The Correlations Between the Subjective AASP and the Psychopathological Symptoms in Both Groups

As reported in Table 8, according to the Pearson correlation test, in children with anxiety, medium to large significant positive correlations were found between the AASP patterns’ scores and anxiety psychopathological symptoms according to the RCMAS: greater sensory sensitivity and sensation avoidance significantly positively correlated with higher levels of anxiety, especially, with the physiological, worry, and over-responsivity, social concern problems, and total anxiety with large effect sizes. Lower registration of sensory input significantly positively correlated with the RCMAS physiological scale and total anxiety score with medium effect sizes.

In the control group, medium to large significant positive correlations were found between the AASP patterns’ scores and anxiety psychopathological symptoms according to the RCMAS: greater sensory sensitivity, low registration and sensation avoidance significantly positively correlated with higher levels of anxiety, especially, with the physiological, worry and over-responsivity, social concern problems, and total anxiety.

Medium significant positive correlations were found between AASP scales and CBCL scales. In the study group: sensation avoidance significantly positively correlated with emotional psychopathological symptoms of withdrawn/depressed, somatic complaints, and internalization. AASP sensation avoidance correlated with worse CBCL psychopathological symptoms of withdrawal/depression, somatic complaints, and internalization. In the control group, greater sensory sensitivity significantly positively correlated with higher anxious/depressed symptoms and somatic complaints; greater low registration significantly positively correlated with higher anxious/depressed symptoms; greater sensation avoidance significantly positively correlated with higher anxious/depressed symptoms and internalization. (See Table 8).

## 4. Discussion

The present study aimed to profile SMDs in adolescents with anxiety disorders and examine their relation to psychopathology. This study used subjective self-reports to reflect SMDs in daily life scenarios from the individual’s own voice, and objective measures to reflect underlying mechanisms that may explain behavioral responses and functional restrictions.

The first hypothesis was confirmed: SMDs were more prevalent among the study group. The AASP subjective self-report found that adolescents with anxiety disorders had higher sensory sensitivity and avoidance, lower ability to register sensory input, and lower tendency to seek sensory input than the controls. A recent meta and retrospective analysis of people with various psychiatric conditions found that based on AASP, SMDs were prevalent and expressed in elevated sensory sensitivity, sensation avoidance, low registration, and a lower tendency to seek sensory input [30,31]. Our results confirm earlier findings according to which SMDs are prevalent among children and adolescents with anxiety disorders [32].

Previous reports which also used both self-reports and objective measures (such as EDA, and functional neuroimaging), were performed on various age groups—from early childhood to adulthood but referred to multiple psychiatric disorders. Tolin et al. (2021) who focused on adults with anxiety disorders used EDA and heart rate recordings in response to auditory startles and found sympathetic hyper-arousability according to both measures. They also found lower autonomic recovery after removing the auditory stressor [18].

Our study focused on anxiety in adolescence and highlighted how important it is to elaborate on the knowledge regarding SMDs, their relation to anxiety severity, and their functional implications in this critical developmental stage. Moreover, our study emphasized the sensitivity of the AASP in demonstrating unmodulated perception of sensory stimuli among adolescents with anxiety disorders. Support for that was found by the objective measures (EDA and heart rate) which depicted higher reactivity and lower adaptation to sensory input. Even though most adolescents in the study group were under medications, they still exhibited an elevated physiological hyper-arousability—in response to the first stressor auditory startle, during the startles’ session (in which adaptation should have been found, in response to the repeated auditory stimuli), and even during the third session of relaxation, emphasizing the lingering effect of sensory stimuli. This information may shed light on daily function obstacles that these adolescents experience. Therefore, hyper-arousability should be evaluated in adolescents with anxiety disorders in order to deeply understand its consequences on patients’ functioning and pathogenesis severity.

With respect to the second hypothesis, SMDs (both sensory sensitivity and sensation avoidance), significantly correlated with anxiety psychopathology severity as manifested in RCMAS results (higher levels of worry and over-responsivity, social concern, and concentration problems) in both study groups. In the study group, the relations with anxiety psychopathology but not with other psychopathological symptoms of the CBCL may be related to the fact that this measure includes behavioral and externalizing symptoms not related to anxiety. Moreover, CBCL was measured according to parents’ reports, while the RCMAS was according to the adolescents’ reports themselves. The relation between SMDs and psychopathology severity was found in studies on other clinical populations. For example, MacLennan, Roach, and Tavassoli (2020) studied 41 autistic children aged between 3 and 14 years using parent and self-reported measures and found that greater sensory hyperreactivity correlated with elevated phobia-related symptoms, total anxiety, and separation anxiety [33]. Interestingly, in the present study, patterns related to sensory hypersensitivity correlated with worse anxiety psychopathology, while sensory seeking correlated with a better clinical profile and more adaptive reactivity and modulation to sensory input. Our results are consistent with prior research on patients with psychiatric disorders indicating that sensory seeking correlated with a better clinical profile and a better quality of life and, therefore, might presumably serve as a protective pattern [34].

The results of the present study support the previous literature highlighting the role of SMDs in anxiety development [35] and psychopathology [6,7]. Studies found that SMDs in adolescents with anxiety disorders are related to nonadaptive behavior [36], worse social functioning [37], and sleep problems [38]. Therefore, SMDs in anxiety disorders should receive clinical and scientific attention [31]. Detailed evaluation of sensory modulation should be performed based on self-reports and objective physiological measures. Non-invasive physiological indicators of autonomic activity [39] such as EDA offer an excellent opportunity to assess and monitor the physiological alterations under different conditions of anxiety [40,41]. Practically, these measures may exhibit clinical utility for aiding clinicians in monitoring the progress of treatment [42,43]. In research, these measures may help in conceptualizing biopsychological processes that can be targeted to optimize care [44]. This detailed information may focus intervention according to the individual unique sensory profiles, unique pathogenesis, and real-life context [34].

In therapy, sensory-based intervention strategies may be relevant for adolescents with anxiety disorders [6,45]. Therapists should elevate the awareness of adolescents, parents, teachers, and health providers to the involvement of SMDs in the pathogenesis of anxiety and on daily function; Applying sensory stimuli that fit the individual’s sensory profile may assist in modulating behavior and function. For example, weighted blankets, Yuckee balls, and wraps may enhance the perception of sensory input to low registrators. For hyper-sensitive individuals, relaxation techniques using sensory-based strategies (such as massage) may be relevant [45]. Sensory modulation intervention may be combined with cognitive behavioral therapy (CBT) and provide strategies to cope with challenging sensory situations in daily life [45].

## 5. Conclusions

SMDs may be prevalent in adolescents with anxiety disorders and explain behavioral hyper-arousablity and functional deficits. SMDs may be related to psychopathological symptoms of anxiety. Since sensory modulation plays a crucial role in the way we perceive the world, connect with others, and function, SMDs should be evaluated in adolescents with anxiety disorders. Evaluations should include self-reports and objective physiological measurements. This may improve intervention outcomes, decrease in the severity of the psychopathological symptoms, and improve function, development, and well-being.

## 6. Research Limitations

The findings of the present study should be interpreted in light of the following limitations: the study groups varied in certain socio-demographic parameters. The study group included various anxiety disorders with different coexistence psychiatric disorders. Therefore, it is recommended to conduct future studies on larger samples with similar socio-demographic characteristics and specific anxiety disorders to enable the generalizability of the results.

Regarding discrepancies identified between the RCMAS and CBCL questionnaires’ correlational results with the AASP, these should be assessed by principal component analysis (PCA) or other multivariate analysis in future studies, using a larger sample.

Finally, most participants in the study group were under psychiatric medication (such as antidepressants), future studies should consider the possible impacts of medications on the results.

## Figures and Tables

**Table 1:** Prevalence of anxiety disorders among the study group.

Psychiatric Diagnosis	Number of Participants (*n* = 44)	Percentile %
Anxiety	8	18.2
Anxiety and ADHD	12	27.3
Anxiety and ODD	2	4.5
Anxiety and depression	5	11.4
Anxiety and adaptive disorder	3	6.8
Anxiety, ADHD and ODD	3	6.8
Anxiety, ADHD, and adaptive disorder	2	4.5
Anxiety, ADHD, and depression	1	2.3
Anxiety, ADHD, and OCD	3	6.8
Anxiety, ADHD, and PTSD	2	4.5
Anxiety, adaptive and borderline personality disorders	1	2.3
Anxiety, depression, and personality disorder	1	2.3
Anxiety, ADHD, and tics	1	2.3

Note: ADHD = Attention deficit and hyperactivity disorder; ODD = Oppositional defiant disorder; OCD = Obsessive-compulsive disorder; PTSD = Post-traumatic stress disorder.

**Table 2:** Sociodemographic characteristics of both groups.

		Control Group (*n* = 62)		95% CIs		Study Group (*n* = 44)		95% CIs	
		*n*	Percentile %	Lower Bound	Upper Bound	*n*	Percentile %	Lower Bound	Upper Bound
	Elementary school	0	0	0	0.058	0	0	0	0.080
	High school	8	12.9	0.066	0.234	10	22.7	0.128	0.37
Mother education	Professional	1	1.6	0.002	0.085	11	25.0	0.145	0.394
	Academic	53	85.5	0.746	0.921	22	50.0	0.358	0.641
	Elementary school	0	0	0	0.058	1	2.3	0.004	0.118
	High school	3	4.8	0.016	0.132	11	25	0.145	0.394
Father education	Professional	6	9.7	0.045	0.195	10	22.7	0.128	0.37
	Academic	53	85.5	0.746	0.921	20	45.5	0.317	0.599
	Below average	2	3.2	0.008	0.110	13	29.5	0.181	0.442
Socioeconomic status	Average	6	9.7	0.045	0.195	10	22.7	0.128	0.37
	Above average	54	87.1	0.765	0.933	21	47.7	0.337	0.620

**Table 3:** Comparing CBCL scores between groups according to the Mann–Whitney test.

	Study Group (*n* = 43) Mean + SD	Control Group (*n* = 60) Mean + SD	Mann-Whitney U	*p*
Anxious/Depressed	10.09 ± 4.7	2.12 ± 2.27	129	<0.001 ***
Withdrawn/Depressed	5.86 ± 3.79	0.92 ± 1.44	264	<0.001 ***
Somatic complaints	5.21 ± 4.54	0.90 ± 1.27	418	<0.001 ***
Social problems	6.14 ± 4.24	0.93 ± 1.56	204.5	<0.001 ***
Thought problems	6.37 ± 4.23	0.75 ± 1.09	217	<0.001 ***
Attention problems	8.88 ± 4.31	2.00 ± 2.53	224.5	<0.001 ***
Rule-breaking	3.65 ± 3.25	0.68 ± 0.98	516.5	<0.001 ***
Aggressive behavior	11.14 ± 6.77	2.40 ± 3.147	250.5	<0.001 ***
Other conditions	5.44 ± 3.41	1.67 ± 1.76	417.5	<0.001 ***
Internalization	68.33 ± 8.37	47.38 ± 7.95	77.5	<0.001 ***
Externalization	60.05 ± 8.66	43.53 ± 8.05	236.5	<0.001 ***
Total problems	65.79 ± 7.77	42.75 ± 10.18	86.5	<0.001 ***

Note: SD = standard deviation; *** *p* ≤ 0.001.

**Table 4:** Comparing RCMAS scores between groups according to MANCOVA and ANCOVA tests.

	Study Group (*n* = 44) Mean + SE	Control Group (*n* = 62) Mean + SE	F _(1,106)_	η_p_^2^		95% CIs	
					*p*	Lower Bound	Upper Bound
Physiological	6.388 ± 0.355	3.289 ± 0.295	41.928	0.289	<0.001 ***	2.149	4.047
Worry and over-responsivity	8.239 ± 0.388	4.863 ± 0.323	41.479	0.287	<0.001 ***	2.336	4.416
Social concern and concentration	4.395 ± 0.269	1.865 ± 0.224	48.466	0.320	<0.001 ***	1.809	3.250
Lie	2.876 ± 0.361	2.781 ± 0.300	0.038	0.000	0.846	−0.871	1.061
Total anxiety score	18.298 ± 0.812	9.724 ± 0.675	61.244	0.373	<0.001 ***		

Note: SE = standard error; η_p_^2^ (partial η^2^) = effect size; *** *p* < 0.001.

**Table 5:** Comparing AASP scores between groups according to the MANCOVA test.

	Study Group (*n* = 43) Mean + SE	Control Group (*n* = 62) Mean + SE	F _(1,105)_	η_p_^2^		95% CIs	
					*p*	Lower Bound	Upper Bound
Sensory sensitivity	46.311 ± 1.599	32.832 ± 1.312	39.266	0.278	<0.001 ***	9.212	17.745
Low registration	39.388 ± 1.473	31.150 ± 1.208	17.282	0.145	<0.001 ***	4.307	12.168
Sensory seeking	41.787 ± 1.101	49.406 ± 0.903	26.483	0.206	<0.001 ***	−10.556	−4.682
Sensation avoidance	40.680 ± 1.408	30.190 ± 1.154	30.701	0.231	<0.001 ***	6.735	14.245

Note: SE = standard error; η_p_^2^ (partial η^2^) = effect size; *** *p* < 0.001.

**Table 6:** Prevalence of SMDs among the study group as compared to the controls according to the AASP, according to chi-square analysis.

	Group	Study Group n %		95% CIs		Control Group n %		95% CIs		χ^2^ (2)	*p*	Fischer
Sensory Model				Lower Bound	Upper Bound			Lower Bound	Upper Bound			
Sensory sensitivity	Below norm	1	2.3	0.004	0.120	12	19.4	0.114	0.308	26.025	<0.001 ***	<0.001 ***
	At norm	13	30.2	0.186	0.451	38	61.3	0.488	0.724			
	Above norm	29	67.4	0.525	0.795	12	19.4	0.114	0.308			
Low registration	Below norm	2	4.7	0.012	0.154	15	24.2	0.152	0.361	21.328	<0.001 ***	
	At norm	25	58.1	0.433	0.716	44	71	0.587	0.807			
	Above norm	16	37.2	0.243	0.521	3	4.8	0.016	0.132			
Sensory seeking	Below norm	19	44.2	0.304	0.589	6	9.7	0.045	0.195	19.082	<0.001 ***	
	At norm	24	55.8	0.411	0.695	50	80.6	0.691	0.885			
	Above norm	0	0	0	0.082	6	9.7	0.045	0.195			
Sensation avoidance	Below norm	2	4.7	0.012	0.154	20	32.3	0.219	0.446	29.457	<0.001 ***	
	At norm	16	37.2	0.243	0.521	35	56.4	0.440	0.680			
	Above norm	25	58.1	0.433	0.716	7	11.3	0.055	0.215			

*** *p* < 0.001.

**Table 7:** Comparing EDA and heart rate between groups according to MANCOVA and ANCOVA tests.

			Study Group (*n* = 37) Mean + SE	Control Group (*n* = 60) Mean + SE	F _(1,97)_	η_p_^2^		95% CIs	
							*p*	Lower Bound	Upper Bound
Phase 2–first startle	Arousability	Response time to 1st stimulus	72.043 ± 0.268	74.757 ± 0.206	59.206	0.386	<0.001 ***	−3.413	−2.013
		Amplitude height—1st stimulus	4.776 ± 0.453	3.372 ± 0.348	5.553	0.056	0.021 *	0.221	2.588
			**Study Group** **(*n* = 43)** **Mean + SE**	**Control Group** **(*n* = 62)** **Mean + SE**	**F _(1,105)_**	**η_p_^2^**			
Phase 2–startles session	Arousability	Time to get to maximum amplitude	147.979 ± 8.909	161.840 ± 7.307	1.338	0.013	0.250	130.304	165.647
		Height of the maximum amplitude	5.952 ± 0.490	4.062 ± 0.402	8.213	0.075	0.005 **	4.980	6.925
	Adaptation	Time to get to minimum amplitude	156.328 ± 9.409	151.482 ± 7.717	0.147	0.001	0.703	137.665	174.991
		Height of the minimum amplitude	3.098 ± 0.314	2.696 ± 0.258	0.903	0.009	0.344	2.475	3.720
Phase 3–relaxation session	Arousability	Time to get to maximum amplitude	268.962 ± 3.567	276.430 ± 2.925	2.423	0.023	0.123	261.887	276.037
		Height of the maximum amplitude	5.303 ± 0.491	3.718 ± 0.403	5.762	0.053	0.018 *	4.330	6.277
	Adaptation	Time to get to minimum amplitude	282.181 ± 3.60	279.326 ± 2.953	0.348	0.003	0.557	275.040	289.323
		Height of the minimum amplitude	3.406 ± 0.396	3.131 ± 0.325	0.260	0.003	0.611	2.616	4.189
Heartrate (phases 1 and 3)	Arousability	Pulse rate at the beginning of the EDA test	90.810 ± 2.372	81.683 ± 1.972	8.126	0.073	0.005 **		
	Adaptation	Pulse rate at the end of the EDA test	88.053 ± 2.11	81.543 ± 1.757	5.205	0.048	0.025 *		

Note: SE = standard error; η_p_^2^ (partial η^2^) = effect size; EDA = Electro-dermal activity; * *p* < 0.05; ** *p* < 0.01; *** *p* ≤ 0.001.

**Table 8:** The correlations between sensory modulation according to the AASP and psychopathological symptoms according to the RCMAS and the CBCL in the study group according to the Pearson correlation test.

			Study Group (*n* = 43)					Control Group (*n =* 62)	
		Sensory Sensitivity	Low Registration	Sensory Seeking	Sensation Avoidance	Sensory Sensitivity	Low Registration	Sensory Seeking	Sensation Avoidance
RCMAS	Physiological	0.531 ***	0.471 **	0.48	0.721 ***	0.581 ***	0.415 ***	−0.085	0.427 ***
	Worry and over-responsivity	0.482 ***	0.278	0.052	0.570 ***	0.679 ***	0.462 ***	−0.149	0.520 ***
	Social concern and concentration	0.544 ***	0.194	0.258	0.536 ***	0.523 ***	0.442 ***	−0.144	0.350 **
	Lie	−0.047	−0.090	−0.044	−0.124	−0.250	−0.180	0.192	−0.118
	Total anxiety score	0.621 ***	0.392 **	0.123	0.731 ***	0.715 ***	0.533 ***	−0.165	0.513 ***
			**Study Group** **(*n* = 42)**					**Control Group** **(*n*= 60)**	
CBCL	Anxious/Depressed	0.147	−0.025	0.132	0.212	0.351 **	0.273 *	−0.066	0.344 **
	Withdrawn/Depressed	0.277	0.226	−0.044	0.482 ***	0.073	0.158	−0.069	0.159
	Somatic complaints	0.236	0.155	0.196	0.309 *	0.289 *	0.227	0.020	0.223
	Social problems	0.074	0.103	0.070	0.167	0.168	0.225	0.066	0.156
	Thought problems	0.150	0.118	0.169	0.167	0.042	−0.006	−0.031	−0.008
	Attention problems	−0.107	0.209	0.125	0.038	0.034	0.101	−0.102	0.083
	Rule-breaking	−0.095	−0.272	0.169	−0.053	−0.030	0.186	0.184	0.090
	Aggressive behavior	−0.086	−0.012	0.089	0.112	−0.050	0.001	0.118	0.059
	Other conditions	0.167	0.029	0.048	0.170	−0.116	−0.067	0.213	−0.089
	Internalization	0.156	0.058	0.074	0.325 *	0.307	0.240	−0.071	0.290 *
	Externalization	−0.041	−0.132	0.099	0.007	−0.035	0.043	0.155	0.105
	Total problems	0.088	0.053	0.124	0.206	0.074	0.091	0.081	0.148

Note: * *p* ≤ 0.05; ** *p* ≤ 0.01; *** *p* ≤ 0.001.

## Data Availability

Data are not publicly available due to privacy and ethical restrictions.
